# Scaling of Floral Display and Mechanical/Hydraulic Support Across Twelve Crabapple Cultivars

**DOI:** 10.3390/plants15162458

**Published:** 2026-08-13

**Authors:** Yongxia Chen, Wen Gu, Peijian Shi, Feixue Jiang, Danyu Zhou, Wangxiang Zhang, Karl J. Niklas

**Affiliations:** 1 Co-Innovation Center for Sustainable Forestry in Southern China, College of Civil Engineering, Nanjing Forestry University, #159 Longpan Road, Nanjing 210037, China; yongxia@njfu.edu.cn; 2 College of Forestry and Grassland, Nanjing Forestry University, #159 Longpan Road, Nanjing 210037, China; gw0815@njfu.edu.cn (W.G.); fxjiang@njfu.edu.cn (F.J.); zhoudy@njfu.edu.cn (D.Z.); 3 School of Integrative Plant Science, Cornell University, Ithaca, NY 14853, USA; kjn2@cornell.edu

**Keywords:** biomass allocation, floral structures, *Malus*, pedicel, scaling analysis

## Abstract

Flowers of the Rosaceae, with their distinct pedicels and well-differentiated floral organs, provide an ideal model for investigating biomass allocation trade-offs associated with the balance between reproductive investment and mechanical/hydraulic support. However, the scaling relationship between supportive structures (e.g., pedicels) and floral display structures (e.g., petals) remains largely unresolved. We examined 12 crabapple (*Malus*) cultivars, measuring their pedicel length, pedicel fresh mass, corolla fresh mass, corolla area, and total flower fresh mass. Scaling relationships among these traits were evaluated using reduced major axis regression protocols. Pedicel fresh mass increased disproportionately with increases in pedicel length, total flower fresh mass, and corolla fresh mass, indicating preferential biomass allocation toward structural and hydraulic support. The scaling exponent between fresh mass of floral organs excluding the corolla (FEC) and corolla fresh mass was lower than unity, suggesting preferential allocation toward floral display structures. Corolla area increased more slowly than corolla fresh mass but showed an approximately isometric relationship with total flower fresh mass, revealing distinct scaling patterns between floral display area and biomass investment. This study provides a cultivar-level perspective on floral support–display coordination, advancing our understanding of reproductive strategies in angiosperms.

## 1. Introduction

Floral morphogenesis and biomass allocation strategies are central to plant reproductive ecology and evolutionary biology because floral structures directly determine pollination efficiency and reproductive success. They also embody adaptations shaped by long-term natural selection acting on pollinator interactions [1,2,3]. These adaptations also reflect natural selection acting on limited resources that lead to trade-offs among different reproductive organs or structures to optimize biomass allocation for reproductive success [4,5,6,7]. Patterns of resource allocation are often gauged by scaling theory, which characterizes nonlinear covariation among organs or traits during growth [5,8,9,10]. Mathematically, these relationships are commonly described by a power-law equation $Y_{2}=\beta Y_{1}^{\alpha }$, where *Y*_2_ and *Y*_1_ are interdependent variables, β is a normalization constant, and α is the scaling exponent of *Y*_2_ vs. *Y*_1_ [8]. When α = 1, the relationship between *Y*_2_ vs. *Y*_1_ is isometric, indicating a proportional one-to-one scaling relationship. In contrast, when α ≠ 1, the relationship is a disproportionate allometric scaling pattern. For example, an increase in leaf area typically requires a disproportionate increase in structural and hydraulic support [11,12], a pattern that has been described as “diminishing returns” because an increase in leaf area, or mass, or any combination thereof generally requires a disproportionately greater investment in leaf lamina or petiole mass [13,14,15]. Thus, a fundamental trade-off exists between structural investment and functional performance in the construction of leaves.

Flowers of angiosperms are complex modular systems that integrate multiple functional components within a compact architecture. Similar to vegetative organs, floral structures require coordinated biomass allocation among supportive components and functionally specialized tissues. However, floral structures differ fundamentally from vegetative organs because their construction is influenced primarily by reproductive functions rather than by physiological functions such as carbon assimilation. Therefore, whether the scaling principles commonly observed in vegetative organs extend to reproductive structures remains an open question. The pedicel serves as both a mechanical and hydraulic structure that connects the flower *sensu stricto* to its subtending stem [16]. Consequently, the pedicel’s morphological traits, such as length, diameter, and biomass, directly influence flower orientation, pollinator access efficiency, resistance to wind or physical disturbance, the ability to transport water, nutrients, and photosynthates to the flower, and the ability to support the development of a fruit [16,17,18]. Similarly, among insect pollinated species, the traits of the corolla, such as size, color, and biomass investment, directly influence pollination and reproductive success [1,19,20]. Likewise, the biomass allocation to non-corolla floral structures, such as the calyx, receptacle, androecium, and gynoecium, collectively profoundly influences reproductive success [2,21]. Surprisingly, however, it remains poorly understood whether consistent rules govern biomass allocation among floral structures or to what extent such patterns are constrained by phylogenetic history or ecological factors. Recent work does however indicate that biomass scaling relationships may differ across biological scales: robust patterns can emerge across species, whereas they may be weak within individual populations of individual species [22]. However, whether and how these scaling relationships are expressed at an intermediate scale, specifically among distinct species within the same genus, has not been comprehensively investigated.

To address this gap, we selected the genus *Malus* (Rosaceae) as a model system. *Malus* provides an ideal model system because of its extensive cultivar diversity, coupled with considerable variation in floral morphology and coloration, which are privileged ornamental value [23]. The floral structure of *Malus* is also highly differentiated, with prominent pedicels and clear distinctions among sepals, petals, stamens, and carpels, thereby facilitating the measurement of morphological traits and biomass. Moreover, *Malus* exhibits substantial intraspecific variation in floral, leaf, and fruit characteristics [24,25,26,27,28], rendering it a useful model system for assessing the robustness and plasticity of scaling relationships under a shared genetic background. In addition, flowering phenology varies markedly among cultivars [29,30]. Early-, mid-, and late-flowering groups are likely exposed to different pollination environments and climatic conditions, leading to a potential divergence in floral organ investment strategies. This variability offers a valuable framework for investigating phenotypic differences and ecological adaptation. Although the floral morphological diversity of crabapples has been well reported, prior studies have usually focused on taxonomic delineation and ornamental trait description [25,28,31,32]. Consequently, quantitative analyses of floral biomass allocation are poorly explored.

The present study investigated the scaling relationships between pedicels and floral organs across 12 crabapple cultivars. We examined the consistency and variation in biomass allocation among pedicels, corollas, and other floral components, and further assessed the relationship between corolla display area and biomass investment. Based on the scaling patterns widely observed in vegetative organs, we hypothesized that floral structures would also exhibit allometric relationships during flower construction. Specifically, we expected pedicel biomass to scale allometrically with floral size, reflecting the balance between mechanical/hydraulic support and reproductive investment. We further expected that the scaling relationship between corolla display area and biomass investment would reveal potential constraints on display investment. By testing these hypotheses, this study aims to clarify how structural support, floral display, and reproductive investment are coordinated during flower construction.

## 2. Materials and Methods

### 2.1. Flower Sampling

**Figure 1:**
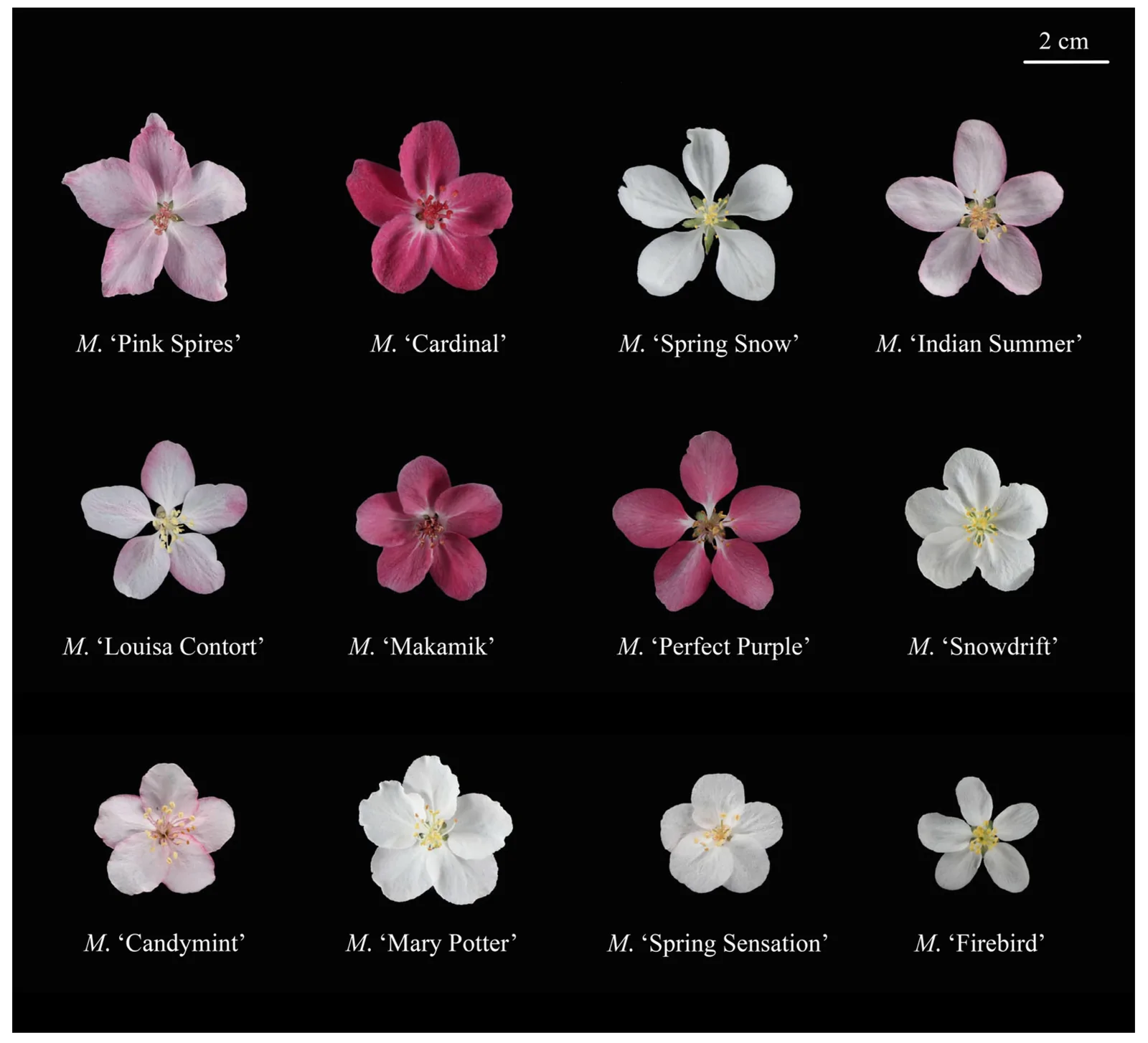
Representative top views of flowers from 12 crabapple cultivars. A scale bar of 2 cm is provided for reference.

**Figure 2:**
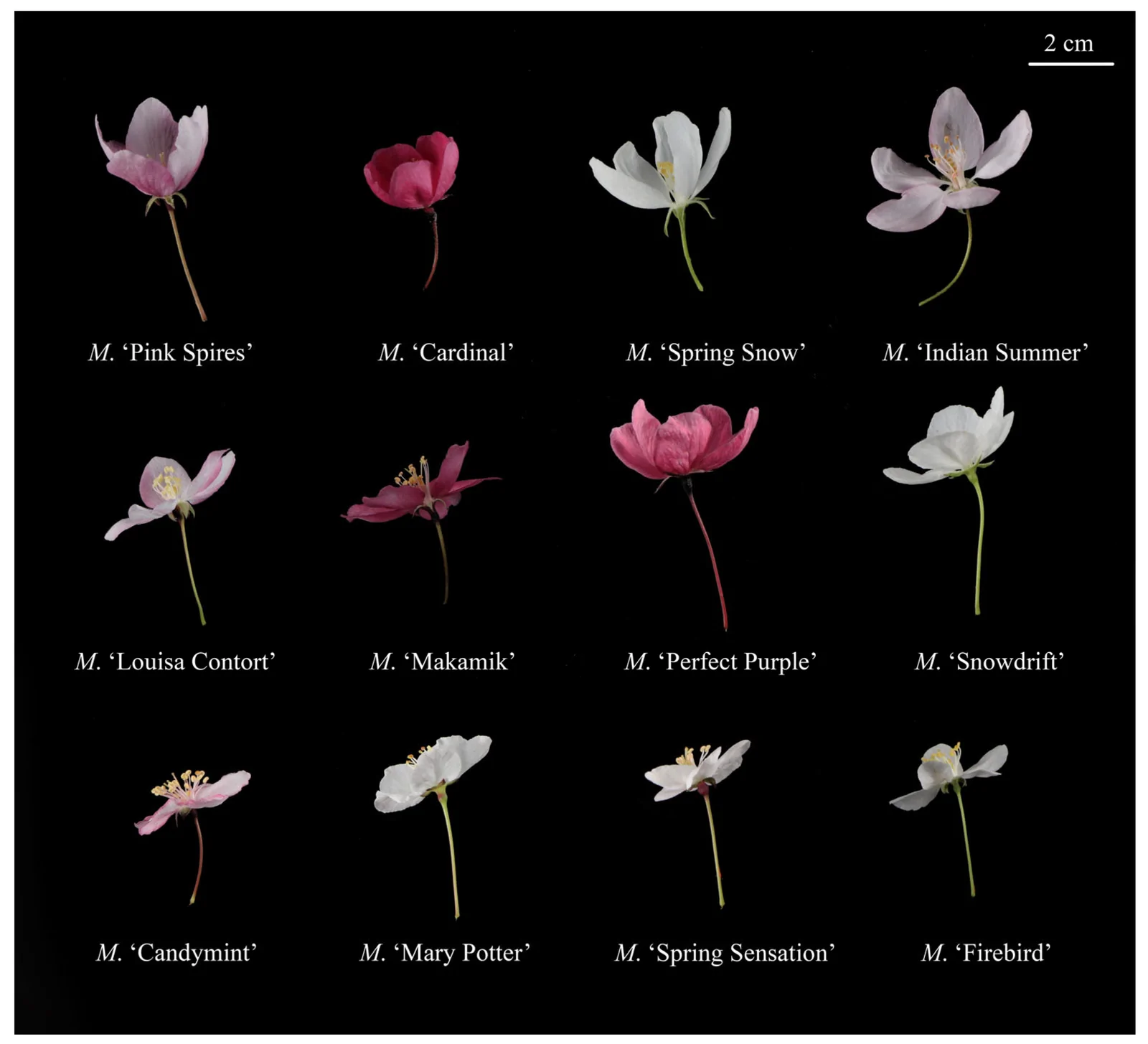
Representative side views of flowers from 12 crabapple cultivars. A scale bar of 2 cm is provided for reference.

Twelve crabapple cultivars differing in floral morphology and flowering phenology, including early-, mid-, and late-flowering types, were selected for this study (Figure 1 and Figure 2; Table 1). Because ornamental crabapple cultivars have complex horticultural backgrounds resulting from extensive hybridization and long-term breeding selection, many cultivars do not have clearly resolved species assignments or complete hybrid formulas. Therefore, the materials in this study were described using recognized cultivar names, following common practice in crabapple germplasm studies [33,34]. The “first flowering” of a cultivar was defined as the date when more than 50% of the observed trees had at least 10% of their flowers open. Flower samples were collected during March to April 2025 from the National Repository of Crabapple Germplasm in Yangzhou, Jiangsu Province, China (32°26′24″ N, 119°39′00″ E). Each cultivar was represented by a planting of 15–20 individual trees. According to climate records from 1980 to 2024 (Source: China Meteorological Administration; https://www.cma.gov.cn/en/; accessed on 1 December 2025), the experimental site experienced a mean annual temperature of 15.78 °C (SD = 0.75 °C) and a mean annual cumulative precipitation of 1273.54 mm (SD = 235.66 mm). For each cultivar, flowering branches with different orientations were randomly sampled from at least 15 individual trees. The excised branches were transported to the laboratory within 5 min after collection and maintained in water-filled containers to prevent desiccation. For all cultivars, only newly opened flowers with fully expanded petals, intact floral organs, and no visible signs of senescence or abscission were selected for measurement. A total of 3707 healthy, fully opened flowers were sampled (with more than 300 flowers for each cultivar).

### 2.2. Measurements of Floral Structures

All measurements were performed under laboratory conditions. Pedicel length (*L*_P_) was measured using a digital caliper (0–150 mm, Shanghai Accurate Measuring Tools Co. Ltd., Shanghai, China; accuracy: ±0.02 mm). Pedicel fresh mass (*M*_P_), flower excluding corolla fresh mass (*M*_FEC_), and corolla fresh mass (*M*_C_) were weighed using an electronic balance (LC-FA1204, Yinzhou Huafeng Electronic Instrument Factory, Ningbo, China; accuracy: 0.0001 g). *M*_FEC_ is the combined fresh mass of the calyx, receptacle, androecium, and gynoecium. Total flower fresh mass (*M*_F_) was calculated as the sum of *M*_FEC_ and *M*_C_. Corolla area (*A*_C_) was measured for 60 flowers per cultivar for six representative cultivars (i.e., cultivars 1, 2, 5, 7, 9, and 10; Table 1) using a photo scanner (V600, Epson Indonesia, Batam, Indonesia). The resulting images were processed in Adobe Photoshop 2021 (version 22.4.2; Adobe Systems Incorporated, San Jose, CA, USA) to extract binary silhouettes of the petal edges, which were saved as bitmap files for subsequent area calculation. To obtain the area of individual petals, the planar coordinates of petal boundaries were extracted using a MATLAB procedure (version 2009a; MathWorks, Natick, MA, USA) developed by Su et al. [35]. The petal area was then calculated using the “bilat” function in the “biogeom” package (version 1.3.5) [36] based on R (version 4.3.1) [37]. The corolla area per flower was calculated as the sum of the areas of all petals per flower.

### 2.3. Statistical Methods

One-way analysis of variance (ANOVA) was used to determine if differences existed in *L*_P_, *M*_F_, *M*_FEC_, and *M*_C_ among the 12 cultivars, and whether differences in *A*_C_ existed among the six cultivars. The coefficient of variation (CV), defined as the standard deviation divided by the mean (in %), was calculated for each trait to quantify intraspecific variation. Post hoc Tukey’s HSD test (with α = 0.05) was used to make pairwise comparisons when ANOVA results were significant (*p* < 0.05).

This study focused on the allocation of biomass between the pedicel and floral organs, and the possible trade-off between corolla area and corolla biomass. All relationships were modeled using the power-law equation between two interdependent variables, *Y*_2_ and *Y*_1_ [8]:

(1) $$Y_{2}=\beta Y_{1}^{\alpha },$$

where α is the scaling exponent of *Y*_2_ vs. *Y*_1_, and β is the normalization constant. To stabilize variance and determine the numerical values of α and β, Equation (1) was log-transformed (using the natural logarithm):

(2) $$\log\left(Y_{2}\right)=\log\left(\beta \right)+\alpha log(Y_{1}),$$

which can be written in the form:

(3) y=γ+αx,

where *y* = log(*Y*_2_), *x* = log(*Y*_1_), and γ = log(β).

Reduced major axis (RMA) regression protocols were used to determine the values of γ and α [8,38], and the bootstrap percentile method with 3000 resamples was used to determine the 95% confidence interval (CI) of α [39,40]. A significant allometric relationship (α ≠ 1) was identified when the 95% CI excluded the value of unity. All statistical analyses were carried out in R (version 4.3.1) [37].

## 3. Results

**Figure 3:**
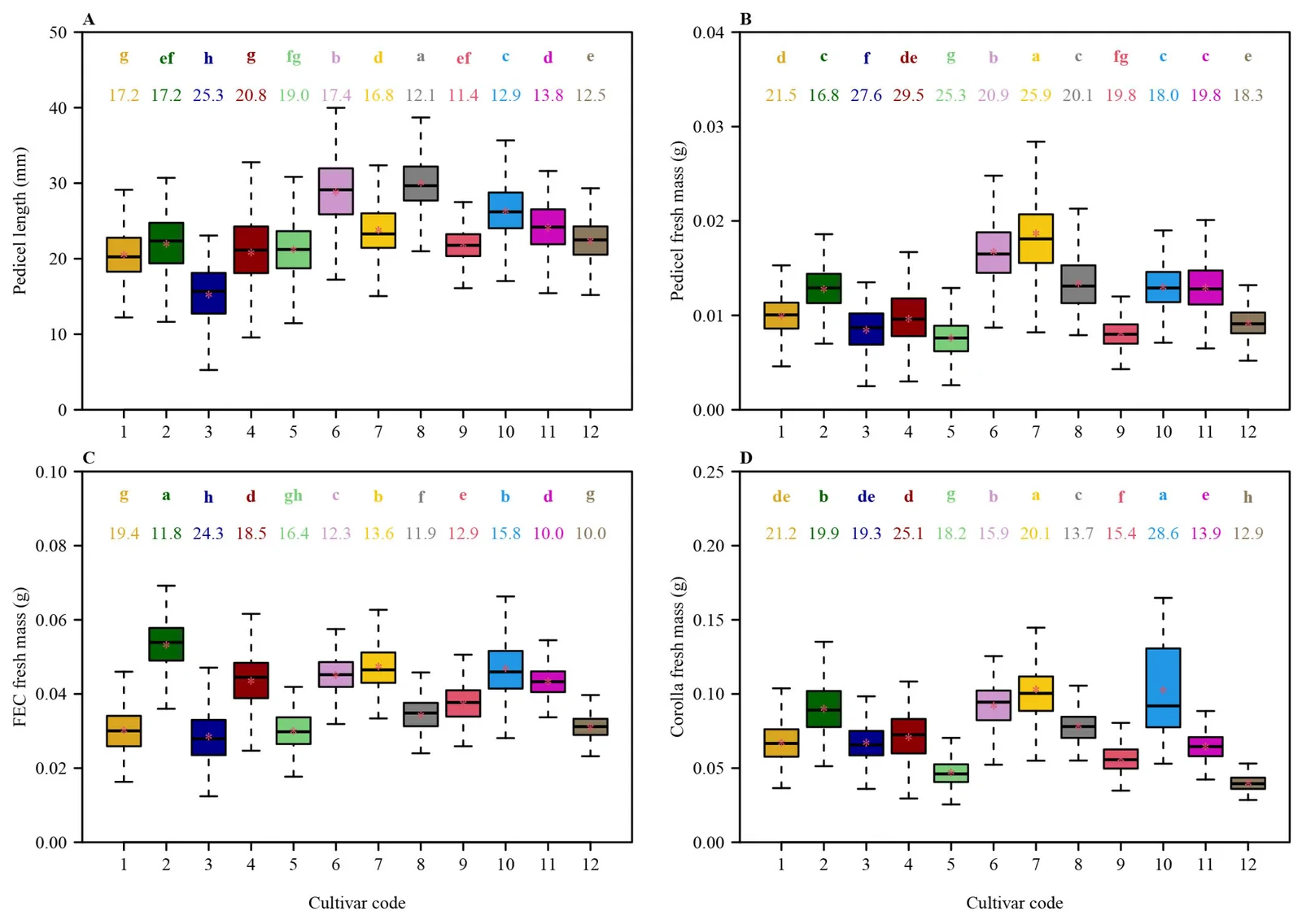
Variation in floral traits among the 12 crabapple cultivars. Boxplots show the distributions of (**A**) pedicel length, (**B**) pedicel fresh mass, (**C**) FEC fresh mass (floral organs excluding the corolla), and (**D**) corolla fresh mass. Different lowercase letters above the boxes indicate significant differences among cultivars, with cultivars sharing at least one common letter not being significantly different according to one-way ANOVA followed by Tukey’s HSD test (*p* < 0.05). The numbers above the boxes represent the coefficient of variation (CV in %) for each trait; the horizontal solid lines represent the medians; and the red asterisks represent the means. Cultivar codes 1–12 correspond to the cultivars listed in Table 1.

There was a significant difference in pedicel length (*L*_P_), pedicel fresh mass (*M*_P_), flower mass excluding corolla fresh mass (*M*_FEC_), and corolla fresh mass (*M*_C_) across the 12 crabapple cultivars (Figure 3). Among the cultivars, *M.* ‘Snowdrift’ had the largest *L*_P_, and *M.* ‘Spring Snow’ had the smallest *L*_P_. *Malus* ‘Perfect Purple’ had the largest *M*_P_, and *M.* ‘Louisa Contort’ had the smallest *M*_P_. *Malus* ‘Cardinal’, *M.* ‘Perfect Purple’ and *M.* ‘Mary Potter’ had the largest three *M*_FEC_ and *M*_C_ (Figure 3).

Analyses of *L*_P_ vs. *M*_P_ indicated that 10 out of 12 cultivars exhibited scaling exponents (α) with 95% confidence intervals (CIs) smaller than unity (Table 2), indicating a significant hypo-allometry, i.e., increases in *L*_P_ do not keep pace with increases in *M*_P_. Notably for two cultivars (*M.* ‘Cardinal’ and *M.* ‘Makamik’), the 95% CIs included unity (0.945–1.116 and 0.817–1.036, respectively), indicating an isometric *L*_P_ vs. *M*_P_ scaling relationship.

**Figure 4:**
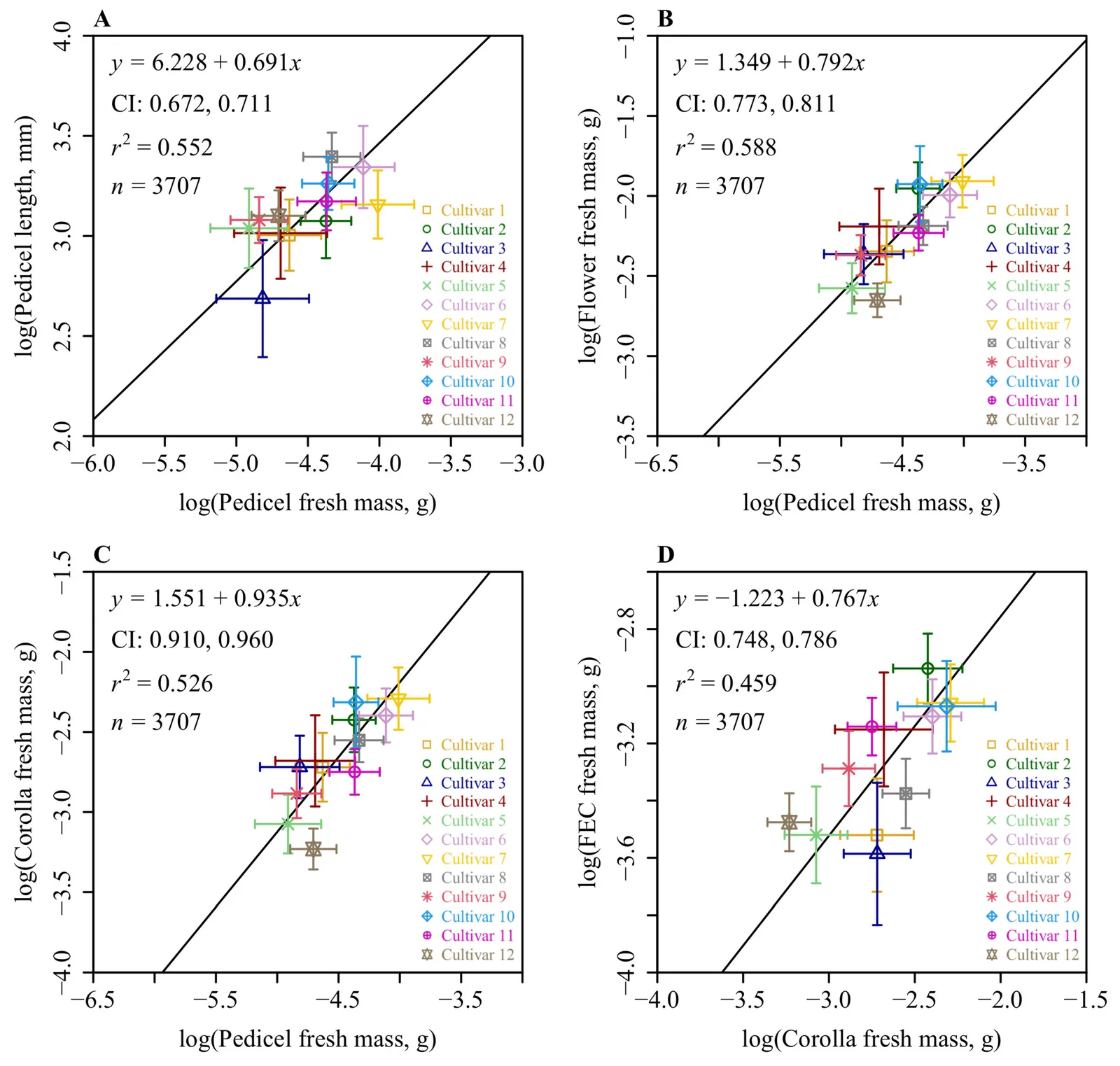
Fitted bivariate relationships of biomass allocation in floral structures across the 12 crabapple cultivars based on pooled data on a log-log scale. There are four scaling relationships: (**A**) pedicel length vs. pedicel fresh mass, (**B**) flower fresh mass vs. pedicel fresh mass, (**C**) corolla fresh mass vs. pedicel fresh mass, and (**D**) fresh mass of floral organs excluding the corolla (FEC) vs. corolla fresh mass. Solid lines represent reduced major axis (RMA) regression lines. Each point and associated error bars denote the mean ± standard deviation of log-transformed values for each cultivar. Each panel displays the fitted power-law equation, the 95% confidence interval (CI) of the scaling exponent, the coefficient of determination (*r*^2^), and the total sample size (*n* = 3707). Data points from different cultivars are color-coded, and the solid line represents the common scaling relationship fitted to the pooled dataset.

Analyses of *M*_F_ vs. *M*_P_ and *M*_C_ vs. *M*_P_ (Table 2) indicated that most of the 12 cultivars exhibited hypo-allometry (α < 1). One such exception was *M.* ‘Mary Potter’, whose α values were significantly greater than unity (for *M*_F_ vs. *M*_P_, α = 1.298, 95% CI = 1.219–1.389; for *M*_C_ vs. *M*_P_, α = 1.563, 95% CI = 1.467–1.675). Similarly, analyses of *M*_FEC_ vs. *M*_C_ (Table 2), hypo-allometry was observed among 10 of the 12 cultivars. The exceptions were *M.* ‘Spring Snow’, which showed hyper-allometry (α = 1.272; 95% CI = 1.153–1.401), and *M.* ‘Louisa Contort’, which manifested *M*_FEC_ vs. *M*_C_ isometry (α = 0.922; 95% CI = 0.837–1.015). Further analysis of the pooled data showed consistent covariation in biomass allocation among different floral structures, with all relationships exhibiting significant hypo-allometry (α < 1; Figure 4).

**Figure 5:**
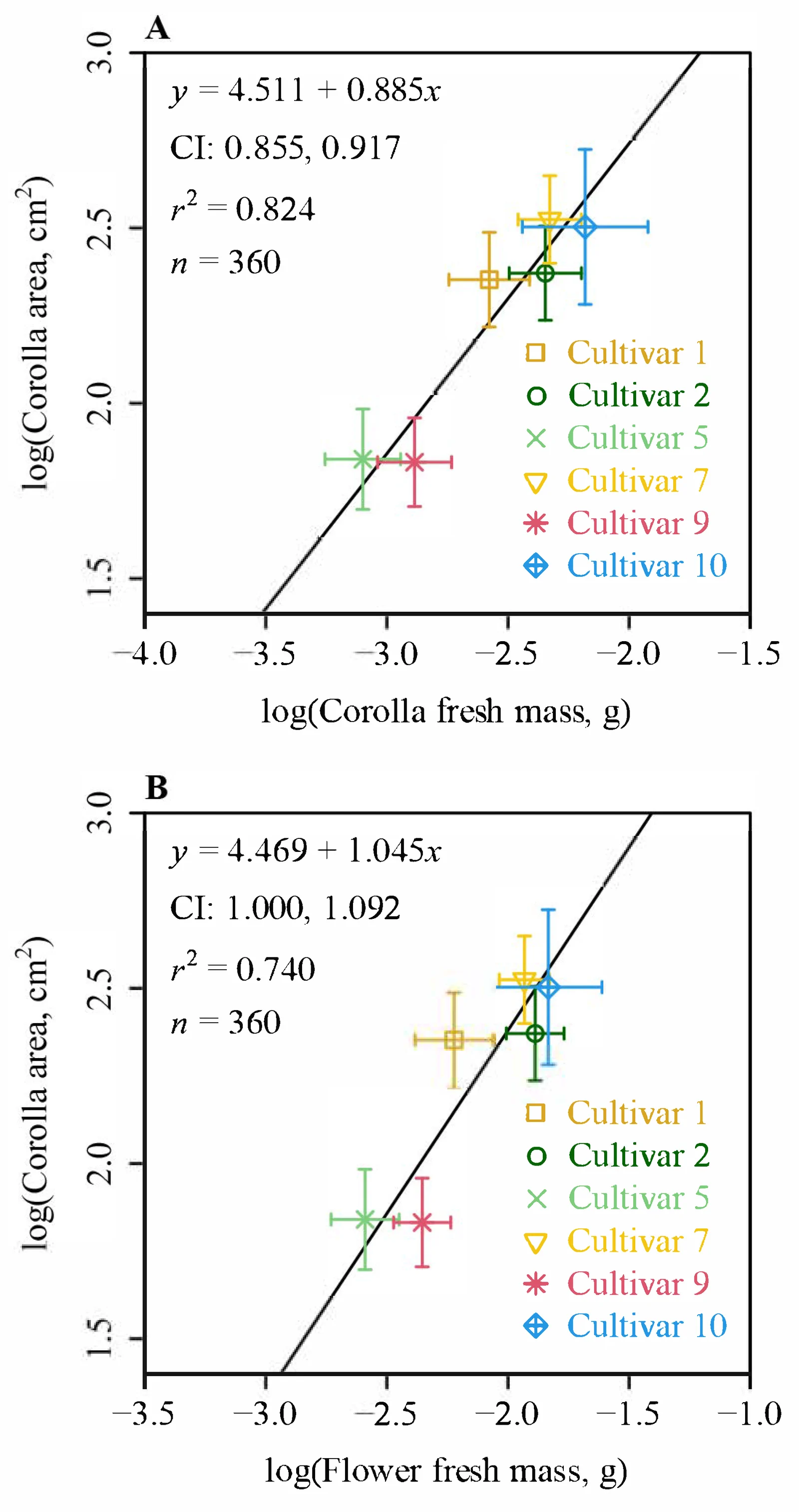
Fitted bivariate relationships of corolla area with corolla fresh mass and total flower fresh mass across the six crabapple cultivars (Cultivars 1, 2, 5, 7, 9, and 10) on a log-log scale. (**A**) Corolla area vs. corolla fresh mass; (**B**) corolla area vs. total flower fresh mass. Solid lines represent reduced major axis (RMA) regression lines. Each point and associated error bars denote the mean ± standard deviation of log-transformed values for each cultivar. Each panel displays the fitted power-law equation, the 95% confidence interval (CI) for the scaling exponent, the coefficient of determination (*r*^2^), and the total sample size (*n* = 360).

Analyses of the scaling relationships of *A*_C_ vs. *M*_C_ and *A*_C_ vs. *M*_F_ among six representative cultivars selected from the 12 cultivars suggested different allocation strategies (Figure 5). In the case of *A*_C_ vs. *M*_C_, the pooled data revealed hypo-allometry (α < 1), i.e., a decrease in display area efficiency with increasing corolla mass. In the case of *A*_C_ vs. *M*_F_, the scaling relationship was isometric (α ≈ 1), i.e., a proportional, one-to-one, display of corolla area with increasing total floral biomass.

## 4. Discussion

This study shows that three critical floral scaling relationships (i.e., *L*_P_ vs. *M*_P_, *M*_F_ vs. *M*_P_, and *M*_C_ vs. *M*_P_) display a phenomenon called “diminishing returns” [13], characterized by scaling exponents (α) smaller than unity. Thus, the biomass allocated to construct the pedicel prioritizes mechanical/hydraulic support over longitudinal extension, in a manner similar to the scaling of lamina mass versus area. Analysis also shows that the scaling of corolla area versus corolla mass in six representative cultivars is hypo-allometric (α < 1), whereas the scaling of corolla area versus total flower fresh mass is isometric (α ≈ 1). Thus, floral ‘display’ (as gauged by corolla area) is maintained despite a “diminishing returns” in structural investment. This observation is strengthened further by the trade-off between total flower fresh mass minus corolla fresh mass (*M*_FEC_) and corolla mass (*M*_C_), which is also hypo-allometric for most of the cultivars examined in this study, i.e., most cultivars prioritize the “construction” of the corolla (possibly to enhance pollinator attraction) with respect to the remainder of their flowers. These findings are discussed in greater detail in the following sections.

### 4.1. Reproductive Strategies Drive Diversity in Floral Biomass Allocation

Prior work has shown that biomass allocation in floral structures can reflect fundamental trade-offs among mechanical stability, pollinator attraction, and reproductive performance [41,42]. In our study, crabapple cultivars exhibited significant differences in pedicel fresh mass (*M*_P_), flower excluding corolla fresh mass (*M*_FEC_), and corolla fresh mass (*M*_C_) (Figure 3). Interestingly, scaling analysis shows that grouping these cultivars based on their flowering time (early-, mid-, and late-flowering) did not explain the differences in biomass allocation among floral components (Table 2), and that some other factor(s) may be responsible (e.g., genetic background influencing floral display or size).

For example, in the scaling relationship between flower fresh mass (*M*_F_) vs. *M*_P_, low scaling exponents occurred in both early-flowering (e.g., *M.* ‘Spring Snow’) and late-flowering cultivars (e.g., *M.* ‘Spring Sensation’). Similarly, both low scaling exponents (α < 1) and high scaling exponents (α > 1) in *M*_C_ vs. *M*_P_ and *M*_FEC_ vs. *M*_C_ were observed across cultivars spanning different phenological groups (Table 2). Based on these observations, we speculate that genetic background and local ecological factors may influence biomass allocation patterns more than flowering time [43,44].

Recent research has shown that the allocation strategy of corolla biomass is directly related to plant pollination efficiency, with larger corollas typically offering greater pollinator attraction [45,46,47]. Our analysis of corolla area (*A*_C_) showed that it scales isometrically with *M*_F_ (Figure 5B; α ≈ 1), indicating that plants maintain a proportional investment in display area relative to their overall floral budget, while simultaneously optimizing display efficiency through the hypo-allometric relationship between *A*_C_ and *M*_C_ (α < 1). Given that petals are transient structures shed after anthesis, this stratagem represents a cost-effective display strategy.

In contrast to the attractive role of the corolla, biomass allocated to non-corolla structures constitutes investment in essential reproductive functions. These non-corolla structures encompass both reproductive organs (e.g., the androecium and gynoecium) and supportive-protective tissues (e.g., the receptacle and calyx). Our findings demonstrate that for most cultivars, biomass allocation to non-corolla components scaled hypo-allometrically with *M*_C_ (e.g., α < 1; Figure 4). The prioritization of the corolla aligns with interspecific patterns where bilateral flowers, often more specialized, invest more in display [48], and with intraspecific patterns where larger flowers disproportionately invest in attractive and reproductive organs [49]. This reflects a strategic prioritization among different functional modules in plant reproductive allocation [50,51,52]. More broadly, these patterns may also be viewed within a source–sink framework, because the development of floral organs depends on the supply and partitioning of imported assimilates [53,54].

### 4.2. “Diminishing Returns” in Pedicel Investment and Its Underlying Mechanism

An integrated analysis of the scaling relationships of *L_P_* vs. *M*_P_, *M*_F_ vs. *M*_P_, and *M*_C_ vs. *M*_P_ shows complex biomass allocation strategies between the supportive structure (pedicel) and the functional display of reproductive organs (floral parts) in crabapple flowers. In the fundamental relationship between *L*_P_ vs. *M*_P_, most cultivars exhibited hypo-allometry with α less than unity, indicating that *M*_P_ disproportionately increases with respect to *L*_P_. From a structure-functional perspective, the pedicel can be modeled as a cylinder with nearly constant tissue density such that its fresh mass (FM = ρ*V*) is positively correlated with the product of diameter squared and length (*V* ∝ *d*^2^*L*). Under conditions of limited longitudinal growth, plants thus preferentially allocate additional biomass to enhance structural strength per unit pedicel length. As a load-bearing organ, the pedicel’s core mechanical property, flexural rigidity (*EI*, where *E* represents bulk tissue elastic modulus and *I* is the second moment of area) [55], is not linearly related to biomass but is determined mainly by morphological structure, particularly the second moment of area [56]. In cylindrical structures, the second moment of area scales with the fourth power of diameter (*I* ∝ *d*^4^), showing high sensitivity to radial dimension [55,57]. It follows that the rapid accumulation of pedicel fresh mass observed in this study most efficiently corresponds geometrically to radial enlargement, indicating a substantial increase in flexural rigidity by optimizing the biomass distribution between petiole length and diameter.

Our analysis further shows that, across most crabapple cultivars, both *M*_F_ and *M*_C_ exhibit hypo-allometric relationships with *M*_P_ (α < 1; Table 2; Figure 4). This pattern indicates that *M*_P_ increases more rapidly than either *M*_F_ or *M*_C_, reflecting a priority for biomass allocation to mechanical or hydraulic support. However, a notable exception to this general trend was observed in *M.* ‘Mary Potter’, which exhibited strong hyper-allometry in both scaling relationships (*M*_F_ vs. *M*_P_, α = 1.298; *M*_C_ vs. *M*_P_, α = 1.563). In this cultivar, flower mass and corolla mass increased disproportionally faster than pedicel mass, suggesting a cultivar-specific allocation tendency toward floral structures, particularly the corolla, relative to pedicel support. This divergence may be associated with its genetic background, floral developmental characteristics, or history of artificial selection for ornamental traits. Future studies across a broader range of cultivars are needed to further clarify the basis of this cultivar-specific allocation pattern.

### 4.3. The Generality of “Diminishing Returns”: From Vegetative to Reproductive Organs

This study suggests a central and therefore potentially important pattern. The relationship between pedicel biomass and the biomass of floral organs generally manifests significant hypo-allometry (i.e., α < 1; Figure 4), which is also seen between the relationship between corolla area and biomass (i.e., *A*_C_ vs. *M*_C_). This phenomenon is similar to the relationship between leaf lamina area and biomass [13] and to patterns observed in the leaf economics spectrum where thicker, denser tissues require disproportionate biomass investment per unit area [12]. Our findings also correspond closely to the scaling patterns observed for other species between petiole and lamina biomass [14,58,59], suggesting that “diminishing returns” may extend beyond vegetative structures and also characterize biomass allocation within flowers.

The similarities among these scaling relationships involve both structural support and the biomass cost of functional area deployment. Petioles and pedicels both provide mechanical support and hydraulic connectivity to the organs they subtend and can be modelled as loaded cantilevered beams [55]. Likewise, leaf laminae and corollas are expanded surfaces whose functional area depends on biomass investment in tissue construction. As these structures increase in size, additional biomass is required to maintain mechanical stability, resource transport, and functional area. The observed relationships therefore reflect a common allocation requirement in which functional expansion must be coordinated with construction and support costs.

However, the biological functions associated with these investments differ substantially. Petioles position photosynthetic laminae for light interception and maintain the mechanical and hydraulic connections required for carbon assimilation [14,60]. By contrast, pedicels support reproductive structures, influence flower orientation and pollinator accessibility, and may persist after fertilization to support developing fruits [16,17,18,61]. Similarly, whereas the leaf lamina primarily functions in carbon acquisition, the corolla serves as a reproductive display involved in pollinator attraction. Thus, biomass allocation within the petiole–lamina system is primarily balanced against photosynthetic returns, whereas allocation within the pedicel–corolla system is balanced against floral display, pollination, and subsequent reproductive performance. The similar scaling outcomes observed in these functionally distinct systems therefore suggest that different biological functions may be constrained by a common requirement to coordinate functional investment with the costs of tissue construction, mechanical support, and resource transport.

### 4.4. Implications and Prospects

From an evolutionary perspective, the recurrence of similar floral scaling patterns at the cultivar level may reflect persistent developmental constraints on floral construction despite hybridization and artificial selection. By analyzing 12 *Malus* cultivars, this study extends floral allometric research to the cultivar scale, complementing previous within-taxon and interspecific analyses. The pooled analysis across cultivars represents an intermediate biological level between variation within individual cultivars and broader interspecific comparisons, allowing common scaling patterns to be evaluated across cultivar diversity. Previous research has shown that scaling relationships are often weak within individual species or populations because of restricted trait ranges, whereas more robust patterns may emerge when broader morphological variation is considered [22]. For example, Wang et al. [22] reported a species-specific coefficient of determination as low as *r*^2^ = 0.079. A comparable situation was observed in the present study, where the relationship of *M*_C_ vs. *M*_P_ in *M.* ‘Spring Sensation’ had *r*^2^ = 0.038. As suggested by Wang et al. [22], such weak within-taxon fits may partly reflect restricted trait ranges, which increase the relative influence of individual biological variation and unavoidable sampling or measurement error. In contrast, the pooled relationships across the 12 cultivars had moderate coefficients of determination (*r*^2^ = 0.459–0.588), including *r*^2^ = 0.526 for *M*_C_ vs. *M*_P_, together with relatively narrow confidence intervals excluding isometry. These pooled analyses therefore provide the primary basis for interpreting the general scaling patterns. Taken together, the cultivar-level analysis links variation among cultivars with common patterns detected in the pooled dataset and shows that the expression and detectability of “diminishing returns” may vary across biological scales.

Meanwhile, several limitations of the present work must be acknowledged. First, crabapple cultivars have undergone long-term artificial selection for ornamental traits, particularly floral display. Therefore, the observed variation and scaling relationships may reflect both horticultural selection history and intrinsic developmental constraints. Comparisons with wild *Malus* taxa would help evaluate the generality of these patterns under natural conditions. Second, although fresh mass reflects the hydrated functional state of flowers and has been used in previous allometric studies [14,62,63], it comprises both dry matter and tissue water. Because pedicels and corollas may differ in water content, part of the observed fresh-mass variation may reflect differences in tissue hydration. Relatively stable differences would mainly affect the intercepts, whereas systematic changes in water content with organ size or across cultivars could also contribute to bias in the estimated scaling exponents. Future studies incorporating dry-mass measurements would help assess the extent of this influence. Third, all samples were collected from a single germplasm repository, which may limit the assessment of phenotypic plasticity and local adaptation potential. Future studies across broader taxonomic and environmental contexts will help clarify the relevance of these patterns to ornamental breeding and plant responses under changing environments. Furthermore, integrating additional functional traits with direct observations of pollinator behavior would more directly link biomass allocation strategies to plant fitness.

## 5. Conclusions

Through an empirical analysis of floral biomass allocation across 12 crabapple cultivars, this study highlighted coordinated but non-proportional scaling relationships among floral components associated with mechanical/hydraulic support, floral display, and reproductive functions. (1) The hypo-allometric relationships of pedicel length (*L*_P_), flower fresh mass (*M*_F_), and corolla fresh mass (*M*_C_) relative to pedicel fresh mass (*M*_P_) indicate that pedicel biomass increased faster than pedicel elongation and floral expansion, reflecting a relative increase in allocation toward mechanical/hydraulic support structures with increasing flower size. (2) The hypo-allometric relationship between corolla area (*A*_C_) and corolla mass (*M*_C_), together with the isometric relationship between *A*_C_ and *M*_F_, suggests that corolla biomass investment is coordinated with overall floral investment but does not translate proportionally into display area expansion. Meanwhile, the hypo-allometric relationship between non-corolla floral organs (*M*_FEC_) and *M*_C_ indicates a relative shift in biomass allocation toward corolla structures compared with other floral components. (3) Overall, this study demonstrates that floral architecture in crabapple cultivars reflects coordinated biomass allocation among support, display, and reproductive components. By extending allometric analysis to reproductive structures, our findings provide quantitative insights into how plants coordinate structural requirements and reproductive functions during flower construction.

## Figures and Tables

**Table 1:** Sampling information of the 12 *Malus* cultivars.

Flowering Type	Cultivar Code	Cultivar Name
Early-flowering	1	*M*. ‘Pink Spires’
	2	*M*. ‘Cardinal’
	3	*M*. ‘Spring Snow’
	4	*M*. ‘Indian Summer’
Mid-flowering	5	*M*. ‘Louisa Contort’
	6	*M*. ‘Makamik’
	7	*M*. ‘Perfect Purple’
	8	*M*. ‘Snowdrift’
Late-flowering	9	*M*. ‘Candymint’
	10	*M*. ‘Mary Potter’
	11	*M*. ‘Spring Sensation’
	12	*M*. ‘Firebird’

**Table 2:** Estimated scaling exponents and coefficients of determination for the scaling relationships between pedicel and floral organ traits across 12 crabapple cultivars. The following scaling relationships were included: pedicel length vs. pedicel fresh mass (*L*_P_ vs. *M*_P_), flower fresh mass vs. pedicel fresh mass (*M*_F_ vs. *M*_P_), corolla fresh mass vs. pedicel fresh mass (*M*_C_ vs. *M*_P_), and fresh mass of floral organs excluding the corolla vs. corolla fresh mass (*M*_FEC_ vs. *M*_C_). Values present the scaling exponent (α) with its 95% confidence interval (CI) in parentheses and the coefficient of determination (*r*^2^).

Scaling Relationship	Cultivar Code	Scaling Exponent (95% CI)	*r* ^2^
*L*_P_ vs. *M*_P_	1	0.802 (0.751, 0.858)	0.728
	2	1.050 (0.945, 1.166)	0.440
	3	0.902 (0.855, 0.955)	0.827
	4	0.700 (0.665, 0.738)	0.857
	5	0.737 (0.685, 0.793)	0.720
	6	0.933 (0.817, 1.036)	0.683
	7	0.671 (0.633, 0.711)	0.763
	8	0.604 (0.566, 0.642)	0.677
	9	0.572 (0.534, 0.612)	0.638
	10	0.719 (0.682, 0.761)	0.731
	11	0.704 (0.658, 0.752)	0.665
	12	0.674 (0.623, 0.727)	0.650
*M*_F_ vs. *M*_P_	1	0.873 (0.796, 0.952)	0.443
	2	0.923 (0.848, 1.008)	0.395
	3	0.578 (0.533, 0.633)	0.589
	4	0.728 (0.692, 0.763)	0.786
	5	0.580 (0.528, 0.638)	0.447
	6	0.635 (0.584, 0.690)	0.381
	7	0.646 (0.595, 0.696)	0.585
	8	0.600 (0.557, 0.645)	0.352
	9	0.630 (0.583, 0.682)	0.453
	10	1.298 (1.219, 1.389)	0.543
	11	0.537 (0.481, 0.603)	0.100
	12	0.558 (0.500, 0.619)	0.247
*M*_C_ vs. *M*_P_	1	0.965 (0.878, 1.057)	0.372
	2	1.141 (1.045, 1.252)	0.324
	3	0.602 (0.546, 0.669)	0.479
	4	0.876 (0.828, 0.927)	0.716
	5	0.679 (0.610, 0.754)	0.369
	6	0.765 (0.701, 0.839)	0.239
	7	0.766 (0.703, 0.830)	0.506
	8	0.680 (0.628, 0.734)	0.274
	9	0.764 (0.700, 0.834)	0.313
	10	1.563 (1.467, 1.675)	0.511
	11	0.693 (0.617, 0.779)	0.038
	12	0.675 (0.603, 0.753)	0.136
*M*_FEC_ vs. *M*_C_	1	0.925 (0.858, 0.996)	0.433
	2	0.604 (0.562, 0.649)	0.570
	3	1.272 (1.153, 1.401)	0.321
	4	0.699 (0.641, 0.760)	0.563
	5	0.922 (0.837, 1.015)	0.283
	6	0.771 (0.702, 0.838)	0.287
	7	0.695 (0.646, 0.749)	0.424
	8	0.893 (0.820, 0.972)	0.338
	9	0.858 (0.786, 0.938)	0.240
	10	0.554 (0.521, 0.587)	0.657
	11	0.707 (0.641, 0.780)	0.244
	12	0.794 (0.738, 0.855)	0.401

## Data Availability

The data can be found in the online Supplementary Tables S1 and S2.

## Supplementary Materials

The following supporting information can be downloaded at https://www.mdpi.com/article/10.3390/plants15162458/s1, Table S1: Fresh mass of the pedicel and floral parts of 12 *Malus* cultivars; Table S2: Corolla areas of six *Malus* cultivars.
